# Exploring the mechanism of empathy on lens language and linguistic landscape on movie-induced tourism: The moderating effect of cultural differences

**DOI:** 10.3389/fpsyg.2023.1109328

**Published:** 2023-02-02

**Authors:** Yanqin Zeng, Ziqi Xu, Liang Chen, Yunxi Huang

**Affiliations:** ^1^Jiangxi Institute of Fashion Technology Media Art Research Center, Nanchang, China; ^2^Department of Art Integration, Daejin University, Pocheon, Republic of Korea; ^3^School of Economics and Management, East China Jiaotong University, Nanchang, China; ^4^Department of Film, Cartoon, and Animation, Cheongju University, Cheongju, Republic of Korea

**Keywords:** movie-induced tourism, lens language, linguistic landscape, empathy, cultural difference

## Abstract

Since the development of film-induced tourism, scholars have increasingly shifted their attention to examining film-induced tourism from different perspectives. However, little research has been devoted to the underlying mechanisms by which audiences empathize with movie scenes. Current research believes that the lens language of movies is helpful for the communication between the movie and the audience. It not only helps the audience to shape the imagination of the movie scene, but also contributes to the construction of a virtual language landscape, and promotes the audience’s cognition of the movie scene. Bringing their emotions and self-expression into the story ultimately enhances the audience’s perception of where it was filmed. In exploring the framework of the transformation of empathy in lens language to landscape language, cultural differences are also proposed as the boundary conditions for the relationship between lens language and empathy. Structural equation modeling with PLS-SEM was employed to test the proposed hypotheses. The findings suggest that lens language positively predicts language landscape and empathy positively mediates the aforementioned relationship. Furthermore, the interaction term of cultural differences amplifies the relationship between lens language and empathy. Finally, we discuss theoretical and practical implications.

## 1. Introduction

Visual centrism considers an important concept of image cognition in Western cultures but has rarely been theoretically tested. With the development of modern streaming media and visualization, visual centrism has begun to invade traditional consumption and affect the customer and tourist experience (Qiu et al., 2018; Li et al., 2021; Zhou et al., 2023). In the process of tourism’s development, the cultural image of tourist destinations has become an important marketing concept, which affects tourists’ attitudes towards destinations and corresponding consumption behaviors in various ways (Li and Katsumata, 2020; Bodhi et al., 2022), For example, tourism planners attract increasing numbers of tourists by constructing chic cultural images for the destinations they manage. In the context of this industry development, the vigorous development of streaming media has promoted the combination of visual centrism and tourism (Busby and Klug, 2001). The integration of visual centrism and tourism promotes film-induced tourism. The original intention of the scene content of the movie in the streaming media is not to attract tourists to the shooting location, but to use the language landscape as the background of the movie to indirectly influence the audience (Wyatt and Badger, 1990). The positive link between movies and tourism is based on the fact that movies can be shown to millions of audiences in a short period, which can leave a good initial impression on moviegoers in tourist destinations (Cao et al., 2018; Yang et al., 2022).

For an existing tourist attraction, a blockbuster movie and a group of well-known actors can bring a great publicity effect (Beeton, 2006; Wu and Lai, 2021). Compared with targeted tourism advertisements and promotions, movie shooting location promotion can influence more target groups with less capital investment and promote tourism consumption (Carl and Smith, 2007; Gong et al., 2020a,b). Hence when tourist destinations lack publicity funds, film-induced tourism is a cost-effective marketing method during economic downturns, and more and more scholars have shown strong interest in research in this field. The success of film-induced tourism stems from the audience’s admiration for the celebrities in the film. In short, because fans want to witness the scenery experienced by their idols, they spend correspondingly on tourism (Yen and Teng, 2015). Some moviegoers yearn for the real scene constructed by the movie, and this image will inspire them to travel (Hudson and Ritchie, 2006). For example, few scholars have argued that film-induced tourism stems from viewers’ perceptions of target images, including the visual language contained in television, film, and other mass media (Connell, 2005). Most of the existing studies on film-induced tourism only focus on the attractiveness of the cultural image of the destination, and there are relatively few studies on how film-induced tourism occurs through audience empathy.

The relationship between tourism and the media comes from the space constructed in the film, such as the shot of the space setting, the narrative setting, and the geographical relationship in the setting (Shiel and Fitzmaurice, 2011). This relationship mimics the concept of lens language defined by Metz (1974). According to this lens, the connection between the tourist destination and the lensed media is likely to stimulate tourists’ expectations about tourism (Polianskaia and Rdu, 2016) and enrich their imagination (Torres and Momsen, 2005). The audience’s imagination is based on the metaphorical expression of the lens language and then turns to the emotional expression of the tourists themselves. In other words, the lens language first affects the audience emotionally, and empathy transforms this physical change into a language landscape, which may stimulate the audience’s motivation to travel through it.

In sum, this study examined the notion of language of the lens in the field of tourism studies through the underlying mechanisms of audience empathy and the results of the linguistic landscape that may connect audiences’ imaginations to tourist destinations. The lens language uses editing and shooting to create emotional metaphors (Zembylas, 2004; Khalid et al., 2021a). We argue that empathy intervenes in the lens language and helps viewers express emotions (Moudatsou et al., 2020), which in turn triggers artificial language landscapes (Anpilova et al., 2020). The interpretation of lens language is not only limited to the narrative technique itself but also seldom examined from the language landscape of the tourism perspective. This study aims to bridge the gap between tourism and film, connect the virtual language landscape imagined by tourists with the lens language of film shooting, realize the audience’s empathy for film-induced tourism, and deepen our understanding of travel motivation, for the follow-up Research on film-induced tourism provides a theoretical reference.

## 2. Literature review and research hypotheses

### 2.1. Movie-induced tourism and linguistic landscapes

With the accelerating pace of life, people have started to enjoy the spiritual realm to overcome and get relief from the pressure of life. Pop culture, with its high commercial value and consumerism, has become a spiritual oasis for people, balancing the efficiency of form with the quality of spiritual enjoyment (Duff, 2002; Khalid et al., 2021b). This change in thinking affects tourism development because, at present, tourism planners are preemptively interested in constructing the destination image by using mass media as a marketing tool to attract tourists, as a combination of visuals and tourism (Beerli and Martin, 2004; Karpovich, 2010). This is in contrast to the traditional tourism approach that focuses on the physical space (Gunn, 1988; Baloglu and McCleary, 1999; Tapachai and Waryszak, 2000). The advantage of mass media-based marketing is that it allows for a quick emotional connection to be built between the tourist and the destination, giving birth to movie-induced tourism (Busby and Klug, 2001; Luqman et al., 2022; Luqman and Zhang, 2022).

Movies attract audiences through vivid and interesting stories and infectious sensory stimulation, which builds audiences’ interest in shooting locations (Chen, 2018). With the movie-induced tourism industry boom, its related theories have become a hot topic of academic research. For example, Evans (1997) argues that “film and television tourism operates in a way that it first appears on the screen and then stimulates tourists to visit these sites and attractions.” Similarly, Riley et al. (1998) defines it as, “the far-reaching influence and spiritual impact on tourists by motivating them to visit locations shown in the shot through the enhanced spectacle of film, television, literature, magazines, records, videos, etc. By reviewing the prior literature, this study defines movie-induced tourism as travel to places by tourists who are attracted by the scenes and locations shot in films. This suggests that the movies strengthen the audience’s knowledge of the travel destination by bringing them a powerful shock to carry out tourism activities toward the shooting location (Evans, 1997; Riley et al., 1998). With the continuous upgrading of the film industry in recent years, movie-induced tourism is gradually becoming a new consumption hotspot in today’s society, and the scenes presented also attract more and more tourists for sightseeing (Ryan, 2009; Luqman et al., 2021; Nusrat et al., 2021).

A language is a human-specific tool used to express people’s thoughts and emotions (Risselada, 2019), while films create an immersive artistic space used to convey virtual feelings (Hahm et al., 2008). They rely on technological means to help those virtual emotions break the limits of physical space and provide a full-sensory experience to the audience (Soler-Adillon and Sora, 2018). This process requires a suitable medium, technology, materials, and artistic language (Carl et al., 2007; Luqman et al., 2020a). The film work creates a new aesthetic space in the interaction process with the audience, which not only transcends human-computer interaction but also achieves the continuation and expansion of physical space. As compared to the rigid insertion of tourism promotion in the film, the linguistic landscape conveyed by the lens language in the film can bring more impact to the audience. Based on this, the study proposes the following hypothesis.

*H1*: In movie-induced tourism, the lens language has a positive effect on the production of tourism linguistic landscapes.

### 2.2. Lens language and emotional metaphors

The film is one of the most dynamic contemporary immersive art forms (Jones and Dawkins, 2018), and its existence draws on digital media such as the language of the camera. Specifically, the film conveys to the viewer, a culture formed by the fusion of the fields of scene perception, event perception, and narrative understanding (Luqman et al., 2020b; Du and Gong, 2022). As an artistic language, lens language works directly on the audiovisual senses of the audience (Metz, 1991), and conveys emotions with visual and concrete images, which has a strong artistic impact (Hao and Ryan, 2013; Bettetini, 2018).

In 2004, the Task Force of the 2nd International Conference on Language and Social Psychology focused on the relationship between language and emerging communication technologies (Hadar and Lian, 2004). They explored how individuals achieve effective alternation through language with or without the presence of various nonverbal cues, which is a core issue in the study of lens structure technologies. This issue is also reflected in topics such as immediate affective expression (Walther, 2012) and the management of online conversations (Foster et al., 2008). With the development of communication systems, system developers have provided new cues and representations of audiences’ emotional expressions (Wuertz et al., 2018; Masood et al., 2020). These are presented in films through the unique design of lens structures, giving audiences a distinctive sense of viewing and emotional attachment. Research addressing lens language in the film will continue to advance the exploration of the core issues of using lense language in other technical contexts.

Current research in the field of lens design technology is still contentious. However, the long-standing debate in this field is mainly related to language’s psychological and communicative impact when people do not communicate directly in person (Walther, 2004). The study of the communicative element of language is reflected in film in terms of the visual perspective, dissecting the structure of shots, and interpreting pictures and films. In other words, the lens language recording of the film shooting includes the cinematographer’s perception of the captured images and the emotions that the director wants to convey to the audience (Bader et al., 2017; Masood et al., 2022). The filmmaker tells the audience a story through the lens, leading them to comprehend the film’s connotations (Bednarek, 2015). The lens language of film often contains an interplay of emotions (Wang and Cheong, 2006). In the study of cognitive linguistics, emotion composition includes cognition and language. Moreover, the form of emotion produced is divided into metaphor and metonymy, where metaphor is regarded as an effective cognitive means of conceptualizing abstract categories (Nie, 2006). Emotion is the most common and important experience in the human life process, there exists an interaction between human cognition and emotion (Kotz and Paulmann, 2011). Emotion is also a very complex and intangible phenomenon, while metaphor is a universal cognitive device that is more ubiquitous (Rickards and Lauren, 2015). Thus, we can say that emotional metaphors are about understanding abstract emotions and experiences through some concrete evidence (Emanatian, 1995; Lubart and Getz, 1997). The lens language of films also contains emotional metaphors, calling the role of emotional metaphors in the lens language as empathy (Kim and Richardson, 2003), which is a technical term in the field of psychology recognized as the ability to identify with and understand the situation of others (Decety and Svetlova, 2012; Decety et al., 2016). This reflects the emotional, cognitive, and conceptual aspects of human connection to achieve mutual recognition (Kerem et al., 2001; Saleem et al., 2021). The audience feels the emotions conveyed by the filmmaker through the lens language, thus triggering their empathy for the film was shooting location. Hence, the study proposes the following hypothesis.

*H2*: In movie-induced tourism, the lens language has a positive effect on the production of the audience’s empathy.

### 2.3. Emotional expression and virtual linguistic landscape

Emotion is an important component of real linguistic communication, and emotional expression can be linguistically connected to studying the subjective components of language (Kunzmann and Richter, 2009). Furthermore, emotional expressions highlight the scientific laws of social practice, and emotions serve as people’s attitudes towards things around them and their activities (Wong and Law, 2017). Therefore, emotional expression can be regarded as a process of stimulating people’s aesthetic psychology and imagination through vivid imagery and language. It also has a very prominent role in the construction process of the virtual linguistic landscape.

Linguistic landscapes are rooted in the connection between people and travel destinations (Katz, 2018). These linguistic landscapes are shaped as a process of understanding the world, expressing ideas, and thus influencing others (Olwig, 1996). At the same time, it allows the ideas to be conveyed more clearly (Burenhult and Levinson, 2008). With time, people not only focus on the material aspect of their needs but also aim at their emotional needs (Stamboulis and Skayannis, 2003; Ning, 2017). Thus in the development of linguistic landscapes, virtual landscapes are created to accommodate the multifaceted needs of tourists (Miklós et al., 2019). Furthermore, the virtual linguistic landscape has high ornamental and artistic value, which breaks through the limitations of temporal and spatial territory. Therefore, it is suitable for viewing, entertainment, and learning (Giordan et al., 2017). Such findings show that the shaping of a virtual linguistic landscape is based on the needs of tourists, and the semiotic language it contains will change with the changing needs of tourists. It is the outward manifestation of tourists’ empathy. Hence, the study proposes the following hypothesis.

*H3*: In movie-induced tourism, the audience’s empathy has a positive effect on the construction of the tourism linguistic landscape.

### 2.4. The mediating role of empathy

Empathy is the ability to map the feelings of others onto one’s nervous system (Lamm et al., 2010). The discovery of mirror neurons suggests that the nervous system can map the observed behavior of others onto its premotor cortex, which is a response to emotional action (Depow et al., 2021). The current definitions of empathy by researchers are divided into the following three main categories: first, Ickes (1993) proposed that empathy is an ability to interpret the thoughts and feelings of others accurately; second, empathy is a mental process in which individuals experience the emotional feelings of others through perception, understanding, and imagination (Singer and Lamm, 2009; Schurz et al., 2021; Zhang et al., 2022). Third, empathy is an ability to experience the intrinsic cognitive-emotional feelings of others based on their perspectives, which results in an emotional state consistent with others (Hoffman, 2008). Moreover, state empathy is a process during information processing that consists of three dimensions: affective empathy, cognitive empathy, and associative empathy (Shen, 2010). Empathy is no longer a single cognitive or affective function but a process consisting of many closely related but distinct states of mind (Khanjani et al., 2015). When individuals activate the subject’s representations of states, situations, and objects, the associated somatic responses are automatically activated (Nummenmaa et al., 2008) is where the role of empathy truly arises. The empathy establishment is conducive to audiences from different cultural backgrounds to interpret the natural scenery and humanistic sentiments presented in the film. This further creates an emotional connection with the filmmakers, allowing emotions to be internalized in the language logic of different cultural backgrounds, thus enabling the audience’s ordinary movie-watching needs to empathize with their consumption needs of tourism (Tucker, 2016).

From the audiences’ perspective, the empathy process is used to describe the emotional connection mechanism integrating the movie-induced industry. While the mediating role of empathy arises mainly due to the change of identity from the audience to tourist, the motivation shift from viewing to travel, and the change of demand from interest to expectation (Yi et al., 2022). In other words, the film footage editing and the camera movement during filming can evoke the audience’s emotional response and promote the audience’s empathy for the film, which is the embodiment of the mediating role of empathy. Thus, the study proposes the following hypothesis.

*H4*: In movie-induced tourism, the audience’s empathy is a mediating factor in the construction of tourism linguistic landscapes influenced by lens language.

### 2.5. The influence of cultural differences

Different individuals internalize society’s norms, rules, and values, and their reflections on society directly influence the individual’s determination of behavior feasibility and rationality (Tyler, 2021). We refer to this shared perception of the social environment as culture, which shapes how individuals behave, interact, and establish relationships with others (Markus and Kitayama, 2010). Cultural differences exist in how people think, influenced by different languages and customs (Heelas, 1984; Heine and Renshaw, 2002; West et al., 2022). The role of cultural differences in communication is often demonstrated by comparing two cultural archetypes, which include individualism and collectivism (Richter et al., 2016). Collectivist cultures are characterized by family integrity, group membership, and strong solidarity, emphasizing interdependence between people (Lam et al., 2009). In contrast, in individualistic cultures, individuals consider themselves independent (Chu and Choi, 2011), with an emphasis on self-reliance, competition, distance from the in-group, and hedonism (Holliday, 2009). Asian cultures such as China and Korea, which have Confucian backgrounds, exhibit higher levels of collectivism, while individualism is more common in Western cultures such as the United States.

Cultural differences may hinder tourism on a superficial level, and the lack of proper communication between scenic managers and tourists may affect the quality of services. But nowadays, more and more travel destinations are consciously exploiting cultural differences to develop their markets. These markets are according to the differences in tourists’ way of thinking, thus enhancing economic and social benefits (Streimikiene et al., 2021). Such reflection is also seen in movie-induced tourism. Suppose the plot of a movie is in line with consumers’ value orientation and can satisfy different cultural needs through abundant movie scenes. In that case, the impact of movie-induced tourism can be maximized. This further shows the importance of the cultural differences of the audience in marketing.

**Figure 1:**
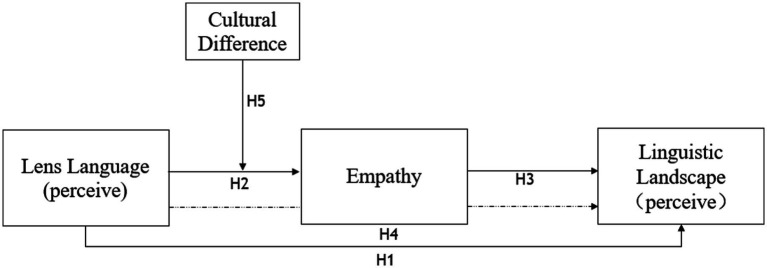
Empathy model in movie-induced tourism.

Culture is also closely related to language. Although culture imposes certain restrictions on the meaning and form of language, language is still the most direct reflection and transmission vehicle, which is also the essence of culture (Varnum et al., 2010). Therefore, the role of language as a mediator is necessary to achieve the transmission of all cultures. Moreover, pragmatic empathy in cross-cultural communication is the ability of people with different cultural backgrounds to communicate emotionally and understand intentions from the other person’s perspective (Ruben, 1976; Gudykunst and Kim, 1984). This study considers the role of pragmatic empathy in culture, determining how the communicative parties can step out of stereotypes brought by culture and avoid conflicts that arise from cultural differences (Young, 2020). Pragmatic empathy is manifested in cultural differences as the two parties of communication respect each other’s cultural background, customs, and intentions of words. Based on this, the study proposes the following hypothesis (Figure 1).

*H5*: In movie-induced tourism, cultural differences influence the contribution of lens language to the tourism linguistic landscape as a moderating variable.

## 3. Research design

We have designed two studies in this article based on the above literature review and research hypotheses. The first study explores the logical relationship between the lens of language and linguistic landscape through the role of empathy. While the second study adds cultural differences as a moderating variable to the first study to investigate the effect of cultural differences on pragmatic empathy in different countries. Both studies considered a survey-based approach to verify the empathy production of the participants. The authors modified the existing established scales in the context and content of this study. The empathy scale is designed drawing from empathy scale of Tian and Robertson (2019), which contains 10 items. Similarly, the film lens language scale is designed based on the study of Wong and Lai (2015) study of celebrity-induced tourism, which selected the dimension of celebrity attractiveness and designed four items. Moreover, the virtual linguistic landscape scale is drawn from Zhang et al. (2015) landscape experience perception scale, selecting two dimensions of the natural landscape and cultural landscape, and designing a total of six items. Lastly, drawing from cross-cultural difference scale of Reisinger and Turner (1998), this study designed the cultural difference scale selecting three dimensions of tourists’ cultural values, social rules, and preferences, resulting in a total of eight items. Likert scales were used to design the questionnaires, where “1” means strongly disagree, and “5” means strongly agree.

## 4. Research 1: Lens language and linguistic landscape under the influence of empathy

### 4.1. Research methods

This study used online collected data methods, considering online professional platforms to distribute and collect questionnaires. We employed PLS-SEM software for the empirical analysis of the data. We first watched random films to ensure the research’s feasibility and provide a basis for the follow-up surveys. Second, we documented the natural and humanistic landscapes recorded by the lens language. Lastly, we conducted data statistics on social media platforms such as Weibo and Xiaohongshu. The results showed that the delicate camera portrayal in the films inspired most tourists to visit the film shooting location to hit the real scene. To ensure the validity and effectiveness of the questionnaire, 10 participants with a greater interest in movies and tourism (on average, they watch movies once a month or travel once a month) were selected for a survey before the formal distribution of the questionnaire. The survey results showed that the participants were impressed by the movies’ natural scenery and humanistic sentiments. Moreover, the metaphorical lens language was no longer enough to meet their spiritual needs, and their willingness to go to the real scene for direct emotional expression gradually increased.

Based on the survey results, we collected participants’ demographic information, taking into account the influence of factors such as gender, occupation, level of education, and other conditions on their interest in movies and tourism. Although a total of 350 questionnaires were distributed in this study, and 331 valid questionnaires were obtained after eliminating the invalid questionnaires with random or missing answers and inconsistencies. The effective recovery rate was 94.6%. Among the overall valid samples of the study, 45.3% were Chinese participants, and 54.7% were participants from other countries. Furthermore, the responses obtained comprised 69.5% males and 30.5% females. The research participants were mainly undergraduate students (accounting for more than 70% of the total), so the sample data were relatively authentic.

### 4.2. Findings

**Figure 2:**
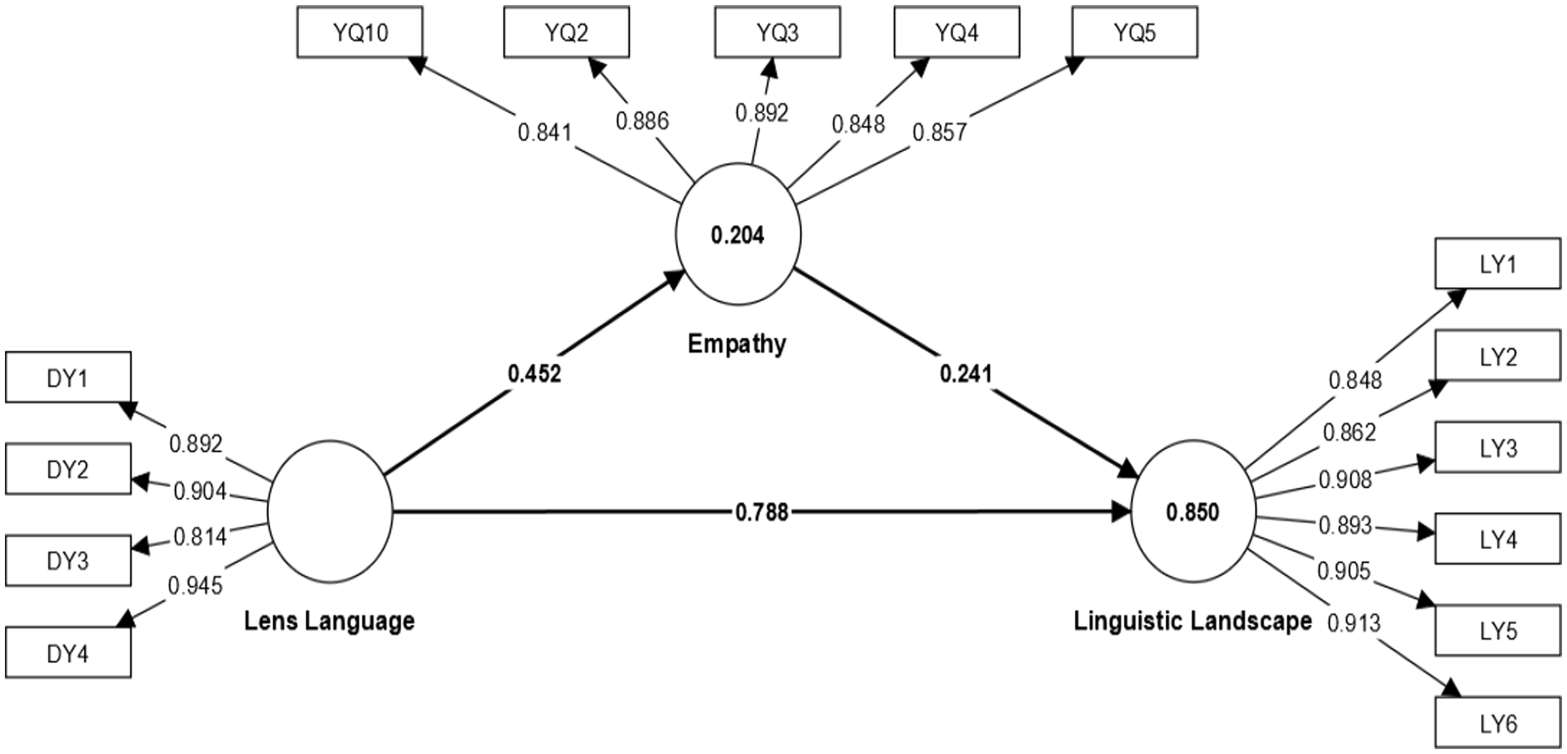
Construction of structural equation model.

Figure 2 presents the results of SEM. The results confirm that the factor loadings for each proposed construct are greater than the 0.6 threshold limit. In addition, we removed certain items due to low factor loadings <0.6, which strengthened the reliability and validity of our proposed scale.

Table 1 showed the explanatory power of the tested models. The *R*^2^ value for the tourism language landscape was 0.850 and the adjusted *R*^2^ value was 0.849. Additionally, empathy had an *R*^2^ value of 0.204 and an adjusted *R*^2^ value of 0.202. The results show that each latent variable has better explanatory power for the tourism language landscape and empathy. In addition, the NFI of the model was 0.685, indicating that the model fit of Study 1 was good.

**Table 1:** Predictive power of the model.

	*R* ^2^	Adjusted *R*^2^
Tourism linguistic landscape	0.850	0.849
Empathy	0.204	0.202

Table 2 presented the standardized values of the reliability and validity of constructs. The Cronbach’s alpha coefficient for the tourism linguistic landscape was 0.947, rho_A was 0.950, the composite reliability (CR) was 0.957, and the average extracted variance (AVE) was 0.789. The Cronbach’s alpha coefficient for empathy was 0.919, rho_A was 0.959, the CR was 0.937, and the AVE was 0.748. The Cronbach’s Alpha coefficient for lens language was 0.912, rho_A was 0.919, the CR was 0.938, and the AVE was 0.792. Our results confirmed that Cronbach’s alpha coefficient of each latent variable is greater than 0.9, the CR is greater than 0.9, and the AVE of each latent variable is greater than 0.5 indicating that each latent variable has good reliability. From the above analysis, it is clear that the model fits well as a whole, the internal latent relationships have significant explanatory power, the estimation effects are all acceptable, and the reliability indicators fit with the structural validity.

**Table 2:** Reliability tests of latent variables.

	Cronbach’s Alpha	rho_A	Composite reliability	AVE
Tourism linguistic landscape	0.947	0.950	0.957	0.789
Empathy	0.919	0.959	0.937	0.748
Lens Language	0.912	0.919	0.938	0.792

The T statistic of each path coefficient was calculated by using the bootstrapping method, and the significance level of the path coefficient was tested (two-tailed test). The specific parameters are shown in Table 3. The T-statistics of the SEM in the Bootstrapping test show that all path coefficients have high T-statistics, with Empathy → Tourism language landscape at 7.435, Lens language → Tourism language landscape at 29.614, and Lens Language → Empathy at 8.103. The value of p of each path is less than 0.05, indicating that the path coefficient is above the corresponding significance level test, and the model structure is stable.

**Table 3:** Significance test results of path coefficients.

	Initial sample (O)	Sample mean (M)	Standard deviation (STDEV)	T-statistic (|O/STDEV|)	*p*-Value
Empathy → Tourism linguistic landscape	0.241	0.242	0.032	7.435	0.000
Lens language → Tourism linguistic landscape	0.788	0.786	0.027	29.614	0.000
Lens Language → Empathy	0.452	0.451	0.056	8.103	0.000
Lens language → Empathy → Tourism linguistic landscape	0.109	0.110	0.022	4.986	0.000

### 4.3. Discussion

Based on the feedback from the above research data and the construction of SEM the following conclusions were drawn.

First, it can be seen through Table 3 that the path coefficient of lens language → tourism language landscape is 0.788, with a *p*-value of 0.000, indicating that lens language has a significant positive influence on tourism linguistic landscape. Indicating that the use of lens language to express film storylines with specific meanings can largely enrich the visual effect of films. And lens language as an artistic language, its strong artistic appeal directly affects the audience’s audio-visual organs, helping the audience to generate strong emotional feedback, and the audience pins this emotion on the tourist language landscape. Hypothesis H1, namely, the language of the shot has a positive impact on the generation of the tourism language landscape in film-induced tourism is verified.

Second, it can be seen through Table 3 that the path coefficient of lens language → empathy is 0.452 with a *p*-value of 0.000, indicating that lens language also has a significant positive effect on empathy, suggesting that under the design of the filmmaker, the editing of the footage gives the film works a rich emotional mindset, shortens the psychological distance of the audience while conveying various sentiment, helps the audience to immerse themselves in the film, stimulates the audience’s empathy for the film shooting place, and promotes their tourism consumption. Hypothesis H2 that the lens language has a positive effect on the production of the audience’s empathy in movie-induced tourism is verified.

Third, Table 3 shows that the path coefficient of empathy → tourism linguistic landscape is 0.281 with a *p*-value of 0.000, indicating that empathy has a significant positive effect on the tourism linguistic landscape. That is to say, the emotional resonance generated by the audience when seeing the film may contain an association with the travel destination, and the established story relationship between characters and scenes in the movie will transform that emotional resonance into a sense of identification with the film shooting location. The hypothesis H3 that the audience’s empathy has a positive effect on the construction of the tourism linguistic landscape in movie-induced tourism is verified.

Fourth, Table 3 shows that the path coefficient of lens language → empathy → tourism linguistic landscape is 0.109 with a *p*-value of 0.000, indicating that empathy plays a significant mediating role in the construction of tourism linguistic landscape by lens language. That is, the audience’s perception of lens language may facilitate the emotional connection with the travel destination, making them anticipate sitting in the movie scene just like the movie characters.

Hypothesis H4 that the audience’s empathy is a mediating factor in the construction of tourism linguistic landscapes influenced by lens language in movie-induced tourism is verified.

## 5. Research 2: Cultural differences influence empathy in movie-induced tourism as a moderating variable

### 5.1. Research methods

Study 2 also employed online methods to collect data, uses online professional platforms to issue and collect questionnaires, and uses PLS-SEM software to conduct an empirical analysis of data. Based on the investigation in Study 1, it was found that cultural differences formed under different education, age, and experience conditions also have an impact on the generation of pragmatic empathy. Accordingly, empathy for cultural differences in language was added as a moderating variable to Study 2.

The revised questionnaire distribution and recovery in study 2, taking into account the impact of language barriers on different nationalities, collected demographic information of different project participants by adding items such as nationality and distributed the questionnaires to participants of different nationalities. A total of 360 questionnaires were distributed, and 334 valid questionnaires were obtained after excluding random or missing or inconsistent invalid questionnaires, with an effective recovery rate of 92.8%. In the entire valid sample, 58.1% were Chinese participants, 41.9% were from other countries, 56.6% were male, and 43.4% were female. More than 70% of the total sample was comprised of undergraduate students.

### 5.2. Findings

**Figure 3:**
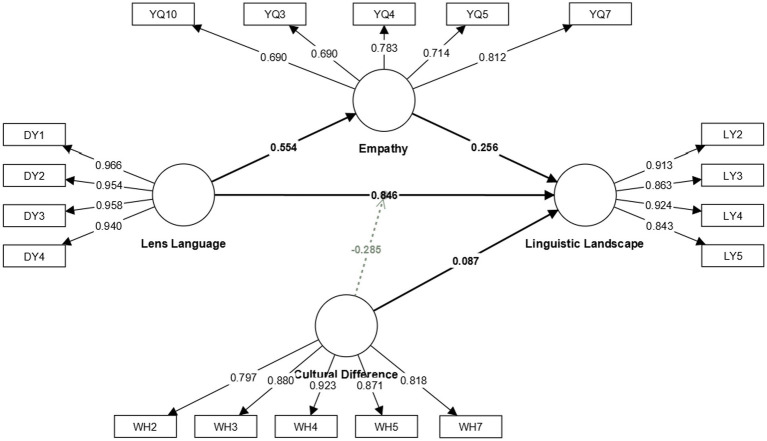
Construction of structural equation model.

Figure 3 presents the results of SEM. The results confirm that the factor loadings for each proposed construct are greater than the 0.6 threshold limit. In addition, we removed certain items due to low factor loadings <0.6, which strengthened the reliability and validity of our proposed scale.

**Figure 4:**
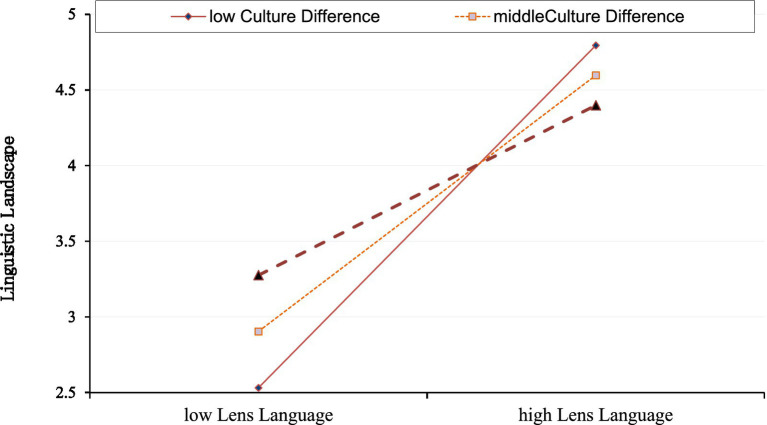
Culture difference as a moderating variable between tourism linguistic landscape and lens language.

Table 4 shows the moderating role of cultural differences in promoting the occurrence of the tourism language landscape through film lens language. Table 4, tested whether cultural differences, as a moderating variable, contribute to the occurrence of tourism linguistic landscapes by film lens language. It is found that the interaction term between lens language and cultural differences positively influences the tourism linguistic landscape with an unstandardized coefficient of −0.285, a *p*-value less than 0.05, and a T-value of 6.536. It shows that cultural differences positively moderate the relationship between film lens language and linguistic landscape until they reach a certain point. Specifically, the stronger the cultural difference, the stronger the impact of lens language on the language landscape, but when this influence reaches a certain level, cultural differences have a negative moderating effect, that is, the lower the cultural difference, the stronger the impact of lens language on the production of language landscape stronger. The details are shown in Figure 4.

**Table 4:** Significance test results of path coefficients.

	Initial sample (O)	Sample mean (M)	Standard deviation (STDEV)	*T*-statistic (|O/STDEV)	*p*-Value
Cultural differences → Tourism linguistic landscape	0.087	0.083	0.040	2.199	0.028
Lens language → Tourism linguistic landscape	0.846	0.843	0.038	22.224	0.000
Lens language → Empathy	0.554	0.557	0.034	16.211	0.000
Empathy → Tourism linguistic landscape	0.256	0.257	0.031	8.248	0.000
Moderation effect 1 → Tourism linguistic landscape	−0.285	−0.283	0.044	6.536	0.000

### 5.3. Discussion

As we expected, the moderating effect of cultural differences on the association between lens language and tourism language landscape in film-induced tourism was confirmed (i.e., H5). In particular, we find that cultural differences actively moderate the contribution of lens language to the language landscape of tourism up to a certain point. After a tipping point or threshold point, cultural differences will have a negative regulatory effect. That is to say when cultural differences have less influence on the audience, the lens language will enhance the audience’s perception of the tourist language landscape when watching the film. Moreover, when the cultural differences of the audience have a greater influence, the lens language will reduce the audience’s perception of the tourism language landscape. Therefore, our findings suggest that cultural differences can be reflected in real-world conditions such as language barriers for communication and inconvenient transportation when traveling, which can reduce audiences’ willingness to travel and reduce their identification with the language landscape.

## 6. Research conclusion

### 6.1. Discussion

Movie-induced tourism is gaining more and more public attention. This paper uses empathy as the intermediary and cultural difference as the moderating variable to explore the impact mechanism of lens language on film-induced tourism and analyze its internal development mechanism. The landscape as a natural visual frame forms a dominant ideologically entrenched set of ideas. However, the landscape presents much more than that. For example, Lefebvre et al. (2004) describes social space as a landscape not just as a place with well-defined physical boundaries, but as a set of relationships, practices, and imaginations. The structure of the camera can not only beautify the language of the scenery but also deepen the artistic creation through the audience’s imagination and actions. Under this specific framework, the film endows the landscape with special connotations and meanings through dramatic conflicts, making it even more mysterious. It also explores how the lens language constructs virtual spaces and how audiences perceive them to realize the connection between film and tourism.

Given the complexity of cultural differences, critical and careful thinking must be considered when examining the impact of cultural differences on such frameworks, both when analyzing the relationship between the two identities of viewer and visitor. Although in common sense, individuals present isolated cases, they can often represent a real region to a certain extent, and can also objectively evaluate the different advantages of different cultures. Therefore, this paper uses empathy as the mediator and cultural differences as moderating variables to explore the language conversion mechanism under film-induced tourism.

Our research finds that the shift from lens language to language landscape does indeed motivate viewers’ willingness to travel. The concept of virtual landscape realizes the collision of imaginary terrain and physical space, making people realize that tourism practice and fantasy can jointly shape living space. For example, under the packaging of a movie, a backward village can become a hotspot, driving the local tourism economy. All the recurring images in the movie scenes have the potential to become popular tourist attractions. This is a trend, and the attraction comes precisely from the language of the film.

### 6.2. Theoretical implications

It is well known that camera language under the influence of empathy contributes greatly to the development of film-induced tourism, so this paper makes the following theoretical findings.

First, the use of language landscapes in tourism research is still novel and rare in the context of cultural tourism (Hao and Ryan, 2013). The lens language is composed of pictures and videos, which provides a new direction for tourism research, that is, to cut into the interpretation of visual factors and conduct in-depth research on tourism.

Second, the lens language of the film enriches the characterization, strengthens the storyline, and attracts the audience through the frame of the film, the setting of the scene, and the alternation of long and short shots. However, the virtual space created by the lens language is immaterial and metaphorical. Pragmatic empathy can well prove the existence of this emotional metaphor and enrich the exploration of the inner mechanism of film-induced tourism.

Third, many studies on film-induced tourism or the relationship between film and tourism are based on cultural identity, where shared values are more likely to generate the same willingness to travel. This article enriches the study of the cultural context in film-induced tourism by addressing cultural differences that emerge before audience intent, examining the impact of different languages and values on empathy.

### 6.3. Practical implications

First, travel and film are both sources of aesthetic material that can engage the public and help people understand and meet the needs of others. Filmmakers can skillfully use the lens language of movie scenes to inspire audiences to engage in a dialog based on the plot of the movie. The lens language is applied to the narrative of the movie, and editing techniques such as montage are used to construct the movie scene. If the scene in the movie is a mainstream tourist destination, the lens language can strengthen the audience’s perception of it; if the scene in the movie is not a mainstream tourist destination, the movie can build a bridge between the audience and new tourist attractions, spark their imagination by forming a special virtual space.

Second, research on lens language interpretation of film shots can help tourism destinations and policymakers understand the complexity of the dimensions of tourists’ imagination. But film tourism destinations are often imagined and reconstructed into surreal scenes by the audience in the film narrative, and sometimes their semiotics or image interpretation will negate the authenticity of the sights, and a correct understanding of the film provides a way to avoid distortion when interpreting the language of the lens.

### 6.4. Limitations and future research

Although this paper contributes to the development of film-induced tourism, it cannot be denied that it still has certain limitations. First, this paper examines the audience’s perception of the lens language in the process of emotional expression and the audience’s attitude towards the virtual landscape. This paper only considers the pragmatic empathy generated by the audience in the cinema, ignores whether tourists who have already gone to the destination are affected by pragmatic empathy, and the scope of research is not broad enough. At the same time, the exploration of the lens language structure is not deep enough, and the feedback on the lens language transformation is only manifested in the audience’s emotional changes, without considering the role of lens structure and lens design variables on the lens language, resulting in insufficient rigor and should be more in-depth. Subsequent research could also examine similarities and differences between the perceptions of linguistic landscapes and tourism virtual landscapes by tourists on film-induced tourism. Second, film-induced tourism-related experiences are all influenced by different societies, cultures, languages, and religions, and the proposed influence of cultural differences was found incidentally during the research process, and the single moderating variable explored is not rigorous enough. Therefore, future research on film-induced tourism should try to introduce multiple moderator variables for in-depth research.

## Data availability statement

The original contributions presented in the study are included in the article/supplementary material, further inquiries can be directed to the corresponding author.

## Author contributions

YZ contributed to the empirical work, the analysis of the results, and the writing of the first draft. ZX, LC, YH, and YZ support the entire work of YZ, and ZX suggests the development of hypotheses. The authors discuss the results and comment on the manuscript. All authors contributed to the article and approved the submitted version.

## Funding

This study was supported by the funding of Jiangxi Province Colleges Humanities and Social Science Project (No. YS22216).

## Conflict of interest

The authors declare that the research was conducted in the absence of any commercial or financial relationships that could be construed as a potential conflict of interest.

## Publisher’s note

All claims expressed in this article are solely those of the authors and do not necessarily represent those of their affiliated organizations, or those of the publisher, the editors and the reviewers. Any product that may be evaluated in this article, or claim that may be made by its manufacturer, is not guaranteed or endorsed by the publisher.
